# Sodium Intake from Foods Exceeds Recommended Limits in the Spanish Population: The ANIBES Study

**DOI:** 10.3390/nu11102451

**Published:** 2019-10-14

**Authors:** Teresa Partearroyo, Mª de Lourdes Samaniego-Vaesken, Emma Ruiz, Javier Aranceta-Bartrina, Ángel Gil, Marcela González-Gross, Rosa M. Ortega, Lluis Serra-Majem, Gregorio Varela-Moreiras

**Affiliations:** 1 Departamento de Ciencias Farmacéuticas y de la Salud, Facultad de Farmacia, Universidad San Pablo-CEU, CEU Universities, Urbanización Montepríncipe, 28925 Alcorcón, Madrid, Spain; t.partearroyo@ceu.es (T.P.); l.samaniego@ceu.es (M.d.L.S.-V.); 2 CIBERESP (Consortium for Biomedical Research in Epidemiology and Public Health), 28029 Madrid, Spain; e.ruiz@externos.isciii.es; 3 National Center for Epidemiology, Carlos III Institute of Health, Avda. Monforte de Lemos, 5, 28029 Madrid, Spain; 4 Department of Food Sciences and Physiology, University of Navarra, Pamplona, 31009 Navarra, Spain; javieraranceta@gmail.com; 5 Department of Physiology, Faculty of Medicine, University of the Basque Country (UPV/EHU), 48940 Leioa, Vizcaya, Spain; 6 CIBEROBN, Biomedical Research Networking Center for Physiopathology of Obesity and Nutrition, Carlos III Health Institute, 28029 Madrid, Spainmarcela.gonzalez.gross@upm.es (M.G.-G.); lluis.serra@ulpgc.es (L.S.-M.); 7 Department of Biochemistry and Molecular Biology II and Institute of Nutrition and Food Sciences, University of Granada, 18010 Granada, Spain; 8 ImFINE Research Group, Department of Health and Human Performance, Universidad Politécnica de Madrid, 28040 Madrid, Spain; 9 Department of Nutrition and Food Science, Faculty of Pharmacy, Madrid Complutense University, 28040 Madrid, Spain; rortega@ucm.es; 10 Research Institute of Biomedical and Health Sciences, University of Las Palmas de Gran Canaria, 35016 Las Palmas, Spain; 11 Service of Preventive Medicine, Complejo Hospitalario Universitario Insular Materno Infantil (CHUIMI), Canary Health Service, Las Palmas de Gran Canaria, 35016 Las Palmas, Spain; 12 Spanish Nutrition Foundation (FEN), 28010 Madrid, Spain

**Keywords:** sodium, salt, consumption, intakes, food groups, Spain, ANIBES

## Abstract

Excessive sodium consumption is associated with adverse health effects. An elevated dietary intake of salt (sodium chloride) has been related to high blood pressure or hypertension, a major but modifiable risk factor for cardiovascular disease, as well as to other ill health conditions. In the present work, our aim was to describe the contribution of foods to sodium consumption within the Spanish population in a representative sample from the “anthropometric data, macronutrients and micronutrients intake, practice of physical activity, socioeconomic data and lifestyles in Spain” (ANIBES) study (9–75 years), to identify high consumer groups, as well as the major food groups that contribute to sodium intake in the Spanish diet. Intakes were assessed by 3-day food records collected on a tablet device. Sodium intakes across the ANIBES study population exceeded recommendations, as total intakes reached 2025 ± 805 mg of sodium per day, that is approximately 5.06 g/day of salt (excluding discretionary salt, added at the table or during cooking). Sodium intakes were higher in males than in females and within the youngest groups. Main dietary sources of sodium were meat and meat products (27%), cereals and grains (26%), milk and dairy products (14%) and ready-to-eat meals (13%). Given the established health benefits of dietary salt reduction, it would be advisable to continue and even improve the current national initiatives of awareness and educational campaigns and particularly food reformulation to decrease overall salt intakes across the Spanish population.

## 1. Introduction

Cardiovascular disease (CVD) is a non-communicable disease (NCD) that is currently the leading cause responsible of death worldwide. It encompasses several conditions such as heart disease, attack and failure, ischemic or haemorrhagic stroke and arrhythmia, amongst others [1,2]. High blood pressure, also known as hypertension (blood pressure ≥ 140/90 mmHg) is the main risk factor for CVD and it has been estimated to cause 17.9 million deaths annually, the equivalent of about 31% of all deaths worldwide [3,4]. Globally, one in four men and one in five women (22% of adults aged 18 years and over) presented high blood pressure (BP) in 2015 [4]. Prevalence of high BP in adults has declined in high-income countries over the last few decades; however, it has been stable or increasing in many low-and middle-income countries [4]. In most European countries, the prevalence of hypertension exceeds 40% [5]. However, these conditions are preventable as they have many modifiable behavioural risk factors such as tobacco use, unhealthy diet and physical inactivity, among others [4]. Excessive sodium consumption is considered the leading dietary risk associated with elevated BP, greater risk of stroke, CVD and premature death [1,4,6]. Evidence shows that reduced sodium intake lowers BP and may prevent hypertension [7]. In addition, an important number of recent high-quality systematic reviews of randomised controlled trials (RCTs) have concluded that decreased sodium intake relative to usual or higher intake, resulted in lowered BP in adults with or without hypertension [7,8,9,10,11]. Independent of BP, further complications are related to excess sodium levels as multiple organs and tissues might be adversely affected: blood vessels, heart, kidneys and the brain [12,13,14].

Salt is the common term used to refer to sodium chloride, which consists of 40% sodium and 60% chloride by weight (5 g salt ≈ 2 g sodium) and it provides about 90% of the sodium in the human diet [15]. Sodium is the principal cation in extracellular fluid in the body, and it is an essential nutrient required for maintenance of plasma volume, acid–base balance, transmission of nerve impulses and normal cell function [16]. Salt is important in food, as a flavour and palatability enhancer, but also for food preservation and safety [17]. In a typical western diet, only 10% to 12% of dietary sodium occurs naturally in foods and the major contributors of dietary sodium/salt intake are processed foods and foods eaten out of home [18,19]. The term “processed foods” includes all foods that have undergone manufacturing methods including not only convenience foods but also products like bread, cheese and meat products [20]. Not surprisingly, in most developed countries, ~80% of salt consumed is added to foods at the stage of manufacturing and in most cases, consumers are not aware of how much salt is added [21]. In non-industrialised countries, the main contributors of daily salt intake (up to 75%) are salt added during preparation, at the table at home, or in condiments (e.g., soy sauce, fish sauce) used for the seasoning of foods in countries such as China [22]. However, in high-income countries like Spain, studies from the Ministry of Health, Social Services and Equality concluded that approximately 70–75% of the salt consumed by our population derives from processed foods and foods consumed outside home, which is also commonly known as “hidden salt” from food as the consumer is not fully conscious of its presence [23]. Sodium is a ubiquitous mineral in the diet, from its presence in a staple product such as bread to a salty snack like potato chips. On the other hand, the sodium content from recipes of home-cooked dishes is highly variable, and discretionary salt use (in home cooking or at the table) is difficult to quantify and often not included in standard dietary surveys [24]. The dietary salt intake among adults in most European countries ranges from 7 to 13 g/day [25]. In 2011, in a representative sample of Spanish young and middle-aged adults aged 18–60 years (*n* = 418), salt intakes were assessed by a 24-h urinary sodium excretion method. Assuming that the sodium eliminated in the urine comes from the diet, sodium excretion corresponded with a dietary salt intake of 9.8 g/day, and it showed that 88.2% of the subjects had salt intakes above the recommended 5 g/day [26]. The World Health Organization (WHO) issued the recommendation to reduce sodium intakes to <2 g/day sodium (5 g/day of salt) in adults, in order to reduce BP and risk of CVD, stroke and coronary heart disease (strong recommendation) [4,5,27,28]. In addition, the WHO recommended a further reduction in sodium intake to control BP in children (strong recommendation), as it should be even lower based on the energy requirements of children [27]. The European Union launched the Framework for National Salt Initiatives in 2008, aiming at a 16% salt reduction within four years across all food categories to achieve the WHO recommendations of an intake of less than 5 g/day of salt (<2 g/day sodium) for adults [29]. In Spain, the Strategy for Nutrition, Physical Activity and Prevention of Obesity (NAOS) from 2005 already recommended that salt intakes from all sources should be also reduced to less than 5 g/day [30]; and since 2008, the government has been actively working towards the reduction of salt consumption. The Spanish Ministry of Health, through the Spanish Agency for Food Safety and Nutrition (AESAN) developed a plan in which first, the average salt consumption from the adult Spanish population was studied, resulting in 9.8 g/day. Secondly, major food salt sources were identified and it was concluded that approximately 70–75% of salt came from processed foods and foods consumed outside the home [31]. Finally, Spanish public health authorities have been closely working with the food industry to establish a framework of collaboration to promote the production and distribution of products which contribute to a healthier and more balanced diet by endorsing a voluntary sodium reduction plan by reformulation of food products [32].

In the present study, our aim was to identify levels of sodium consumption derived from foods, its main food group sources from the Spanish diet and anthropometric characteristics from different population groups from the ANIBES study.

## 2. Materials and Methods

The complete design, protocol and methodology of the “anthropometric data, macronutrients and micronutrients intake, practice of physical activity, socioeconomic data and lifestyles in Spain” (ANIBES) study have been described in detail elsewhere [33] and references are fully available at the repository from the Spanish Nutrition Foundation (FEN) http://www.fen.org.es/anibes/es/biblioteca. The ANIBES study is a cross-sectional study conducted using stratified multistage sampling. The fieldwork was completed at 128 sampling points across Spain. The design of the study aimed to define a sample size representative of all individuals living in Spain, aged 9–75 years, and living in municipalities of at least 2000 inhabitants. The initial potential sample consisted of 2634 individuals, and the final sample involved 2009 individuals (1013 men, 50.4%; 996 women, 49.6%). In addition, for the youngest age groups (9–12, 13–17 and 18–24 years), a “boost sample” was included to provide at least *n* = 200 per age group (error ± 6.9%). The random sample plus boost sample comprised 2285 participants. The sample quotas according to the following variables were: age groups (9–12, 13–17, 18–64 and 65–75 years), gender (male/female), geographical distribution (Northeast, Levant, Southwest, North–Central, Barcelona, Madrid, Balearic and Canary Islands) and locality size (2000 to 30,000 inhabitants being rural population; 30,000 to 200,000 inhabitants being semi-urban population; and over 200,000 inhabitants being urban population). In addition, other factors for sample adjustment were considered: unemployment rate, percentage of foreigners (immigrant population), physical activity level and education or economic level. The fieldwork was conducted from mid-September 2013 to mid-November 2013, and two previous pilot studies were also performed. To equally represent all days of the week, study subjects participated during two weekdays and one weekend day. The final protocol was approved by the Ethical Committee for Clinical Research of the Region of Madrid, Spain [34].

Study participants used a tablet device (Samsung Galaxy Tab 27.0, Samsung Electronics; Suwon, Gyeonggi-do, South Korea) provided by the researchers, to record all food and drinks consumed for three days by taking photos both at home and outside. No specific protocol was given to subjects regarding consumption of their meals, so they would eat as normally as possible. Pictures of dishes or food products were freely taken by participants before and after each eating occasion, with the tablet or a digital camera A specific software was developed to collect information from the field tablets every two seconds, and the database was updated every 30 min. Food consumption was assessed with the use of the photographs, descriptions and collected information, by a team of dieticians/nutritionists who codified the foods and beverages and assigned grams following three different cleanings of the data. Food and beverage sodium contents were calculated from the food consumption records using VD-FEN 2.1 software, a dietary evaluation program from the Spanish Nutrition Foundation (FEN), Spain, which was also newly developed for the ANIBES study based on the Spanish Food Composition Tables [35], with several expansions and updates. Data obtained from food manufacturers and nutritional information provided on food labels were also included. Results are expressed as grams of sodium and discretionary salt use (salt added during cooking or at the table) was not estimated.

Trained interviewers collected the different anthropometric measurements following the procedures previously tested in two pilot studies. Height was measured by triplicate using a Stadiometer model Seca 206 (Seca, Hamburg, Germany), weight was acquired by one determination in a weighing scale model Seca 804 (Seca, Hamburg, Germany). Finally, waist circumference was measured by triplicate using a tape model Seca 201 (Seca, Hamburg, Germany). 

Values are reported as means ± standard deviation (SD) or as percentage. The Kolmogorov–Smirnov test was used to establish if the samples were parametric or non-parametric. Non-parametric data were analysed by the Mann–Whitney U test or the Kruskal–Wallis test. When it resulted in differences, multiple comparisons between medians were studied by the Dunn test to adjust for multiple comparison and adjust the *p*-value with Bonferroni correction. Differences were considered significant at *p* < 0.05. Data analysis was achieved with SPSS 24.0 software package (IBM Corp., Armonk, NY, USA).

## 3. Results

Sodium intakes derived exclusively from foods from the entire population and different gender and age groups are shown in Table 1. Male participants had significantly higher sodium total intakes (2218 ± 868 mg/day) than females (1828 ± 682 mg/day) regardless of age (*p* < 0.05). Furthermore, average daily sodium intake was highest among adolescents aged 13 to 17 years (2351 ± 841.8 mg/day) and children aged 9 to 12 years (2247 ± 735.4 mg/day) although only a small and non-significant variation was observed between them (Table 1). However, significant differences (*p* < 0.001) were observed amongst children and adolescent sodium intakes when compared to adults (2026 ± 805.2 mg/day) and the elderly (1693 ± 640 mg/day).

Table 2, which summarises sodium intakes exclusively derived from foods per 1000 kcal consumed, showed that there were no significant differences per gender within different age groups. Nonetheless, it can be observed how sodium consumption decreased significantly with age. In particular, sodium intakes per 1000 kcal were significantly higher among younger population groups (children and adolescents) than among the elderly (*p* ≤ 0.05).

When studying different eating occasions (Table 3), average breakfast sodium intakes were higher for children (376.4 ± 188.6 mg/day); adolescents presented a higher morning break (278.6 ± 335.6 mg/day) and dinner intake (824.9 ± 487.9 mg/day), while adults had a greater consumption over lunch (692.6 ± 406.3 mg/day), although these differences were not significant. Likewise, there were no significant differences in sodium intakes considering if consumption over the week or the weekend.

**Figure 1:**
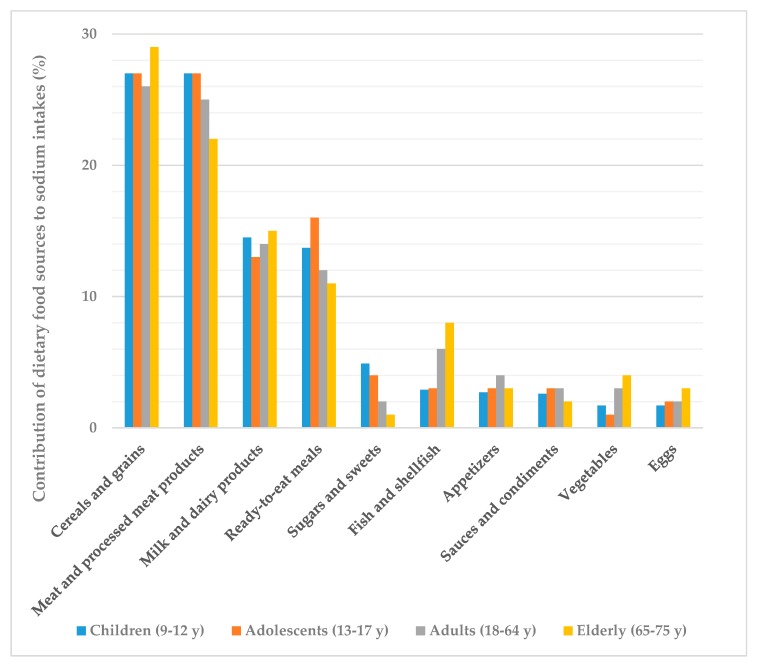
Dietary top ten food and beverage groups contributing to sodium intakes by different age groups from the ANIBES study population.

The main sources of sodium intakes across all groups from the ANIBES study population were meat and meat products (27%) and cereals and grains (26%), followed by milk and dairy products (14%), ready-to-eat meals (13%) and fish and shellfish (6%) as shown in Figure 1. Altogether, these five food groups accounted for more than 80% of sodium consumption. Only those foods that contributed at least 1% to sodium consumption of the population have been included.

When assessing specific food categories within each food group we found that largest contributors to sodium intakes were sausages and other processed meat products (403.8 ± 371.9 mg/day), closely followed by bread (400.4 ± 244.4 mg/day), then ready-to-eat meals (261.9 ± 330.2 mg/day), cheeses (150.0 ± 209.2 mg/day) and canned fish and shellfish (74.1 ± 182.3 mg/day) (Table 4). 

It is noteworthy that meat and processed meat products contributed to sodium intakes in the same proportion as cereals and grains amongst children and adolescents (27%) (Figure 1). Another major food group for adolescents were ready-to-eat meals, which accounted for 16% of sodium intakes. Milk and dairy products contributed very similarly to sodium intakes within all age groups (13–15%) but fish and shellfish showed a higher consumption amongst adults (6%) and, specially, the elderly (8%). The rest of food groups were minor contributors to sodium dietary intakes as they provided 4% or less each.

Subjects were divided into four groups by gender: underweight, normal weight, overweight and obese groups, according to BMI recommended by WHO for children [36] and adults [37]. Considering BMI values, as shown in Table 5, children who were overweight showed significantly higher sodium intakes than those who were underweight. However, no significant differences were observed related to waist/height ratio and cardiovascular risk and age segment [38].

**Figure 2:**
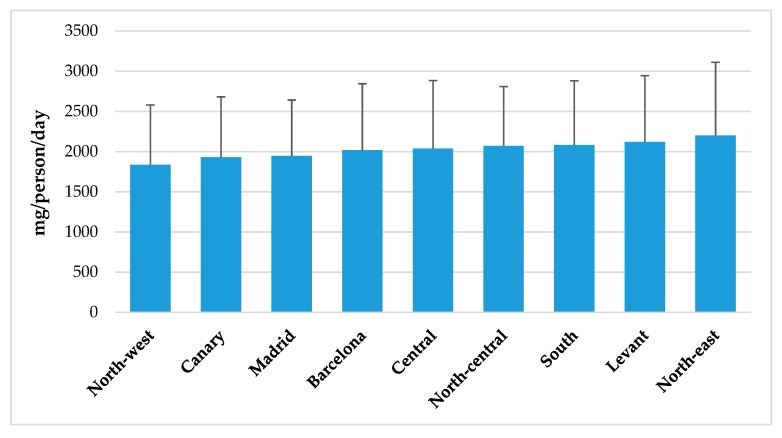
Sodium intake from total population segmented by Spanish Nielsen areas from the ANIBES study population.

**Figure 3:**
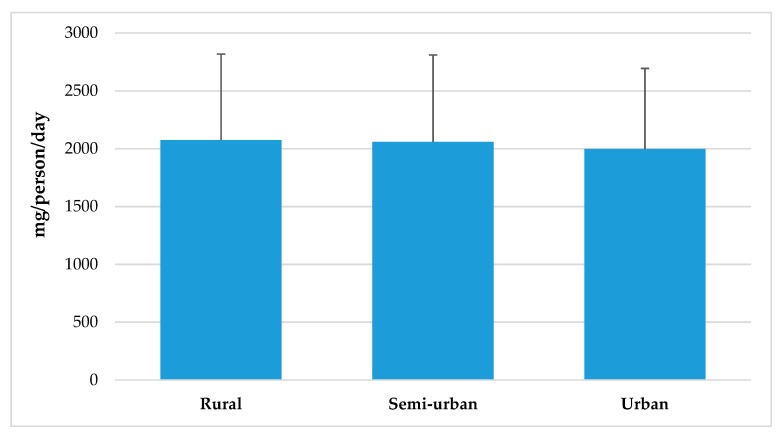
Sodium intake from total population by habitat size from the ANIBES study population.

**Figure 4:**
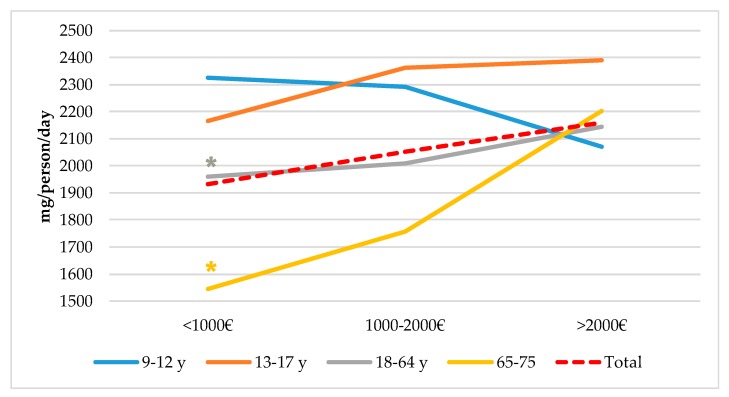
Sodium intakes by income level and age groups from the ANIBES study population. * *p* < 0.05 compared to >2000€ (Kruskal–Wallis test and the Dunn test to adjust for multiple comparison and adjust the *p*-value with Bonferroni correction).

Figure 2 represents sodium intakes from total population segmented by Nielsen zones. Although there were no significant differences between geographic zones in Spain, higher intakes can be observed in South, East and North-East regions when compared to consumption levels from North-West, and Canary Islands areas. Likewise, habitat size (Figure 3) does not seem to exert an influence in population’s sodium intakes, even though there is a tendency to be slightly higher in rural vs. urban areas. However, we observed a different sodium intake between adult (18–64 y) and senior (65–75 y) groups and between lower (≤1000€) and higher (≥2000€) income levels. In both cases, higher sodium consumption (*p* < 0.05) was related to population groups from higher income levels (Figure 4).

## 4. Discussion

The current examination of nationally representative data presents an updated insight into sodium intakes by age group, gender, eating occasions, geographical area, income level and body weight from the ANIBES Spanish population. Furthermore, we identified the major food sources of sodium in the Spanish diet, excluding added salt.

### 4.1. Sodium Intakes by Gender and Age, Total and Per 1000 kcal

Sodium intakes across the ANIBES study population exceeded recommendations, as total intakes reached 2025 ± 805 mg of sodium per day, which is approximately 5.06 g/day of salt, compared to the WHO recommendations of ≤ 2000 mg/day of sodium and 5 g/day of salt for adults [27]. Men consumed higher quantities than women, regardless of age. These differences may be attributable to the higher amount of foods consumed by males, as they fade when sodium consumption is expressed in mg/day per 1000 kcal (1130 ± 307 mg/day for males and 1106 ± 322 mg/day for females).

Previous research has already shown a concerning excessive sodium intake within the Spanish population. Ortega et al. [26] studied the sodium intake of a representative sample of Spanish young and middle-aged adults aged 18–60 years (*n* = 418) by means of the 24-h urinary sodium excretion method, which is considered the gold standard for estimating sodium intake [39]. Authors reported a dietary salt intake of 9.8 (SD 4.6) g/day (3920 mg/day of sodium), and that 88.2% of the subjects had salt intakes above the recommended 5 g/day. They also found a higher salt intake in males and in participants with higher BMI. These values are much higher than our results, which may be explained in part by the different sodium assessment methodology that assumes all urinary sodium comes from the diet. In addition, these differences may account for the discretionary salt use, which is not quantified in our study and potential underestimation of other sodium food sources. Moreover, reformulation policies to lower salt content in foods (e.g., bread) have been in constant progress and successfully implemented in our country during the last years.

The Global Burden of Disease (GBD) study appraised that a diet may be considered high in sodium when it results in an average 24-h urinary sodium excretion higher than 3 g/day [3]. In 2017, the GBD study estimated that in the 28 European Union (EU) member states, a diet high in sodium was responsible for more than 182,000 deaths and 2,950,000 disability adjusted life years (DALYs), both mainly associated with CVD, stomach cancer and chronic kidney disease [3]; therefore, sodium intake reduction has become a priority in public health policies [40].

According to the European Commission (EC) data from 2013, salt consumption amongst adults in most European countries ranged from 7 to 13 g/day (2800–5200 mg sodium/day); therefore, exceeding WHO recommendations. Germany, Cyprus, Bulgaria and Latvia reported the lowest salt intake (6.3–7.3 g/day), whereas the Czech Republic, Slovenia, Hungary and Portugal presented the highest salt intake (12.3–13.6 g/day) [40]. Nonetheless, lower salt consumption was found amongst the Austrian population, where the mean dietary salt intake in adults was 5.6 g/day (2240 mg sodium/day) [18], which is in line with our results. 

Of major concern are the results that indicate that sodium intakes were significantly higher amongst children and adolescents when compared to adults and the elderly. These differences imply that, in our study population, children and adolescents are the specific population groups that primarily exceed the WHO recommendations and public health campaigns and policies should be targeted at them as a high intake of salt is linked to the risk of early development of cardiovascular risk factors such as hypertension [41]. Aparicio et al. [42] assessed sodium intakes from a sample of 205 Spanish schoolchildren aged 7–11 y by measuring 24-h urinary sodium excretion. They found that 84.5% of subjects aged ≤ 10 y had intakes of >4 g salt/day, and 66.7% of those aged >10 y had intakes of >5 g salt/day. These findings indicate only slightly higher sodium intake levels than our results, regardless of the different methodologies used. The US National Health and Nutrition Examination Survey, 2011–2012 (NHANES) evaluated a nationally representative sample of 2142 children aged 6 to 18 years who completed a 24-h dietary recall. Their results reported even higher levels of average daily sodium intake among adolescents aged 14 to 18 y (3565 ± 120 mg) [43]. 

### 4.2. Sodium Intakes from Foods (Excluding Added Salt) Consumed at Different Eating Occasions by Age Groups

Regarding dietary patterns from the ANIBES study population, examination of sodium intakes over different eating occasions showed no significant differences across gender or age groups, neither did week or weekend consumption. However, we did observe that dinner accounted for 30–37% of total sodium daily intakes, being the highest contributor amongst eating occasions, since lunch provided 25–34%, while morning and afternoon breaks provided only 5–11% of total sodium intakes. It is important to remember that dinner is the most common eating occasion to be consumed at home, meaning that personal and family responsibility should be addressed. In addition, this is the first time that sodium intakes are evaluated by eating occasions in Spain. Dickinson et al. [44] studied these patterns in a nationally representative survey in Australia amongst an adult population (18–84 y, *n* = 7818) and found that dinner and between-meal-time eating occasions were the main contributors to total sodium daily intakes (33% and 20%, respectively). Similar results were derived from the US NHANES for children aged 6 to 18 years, where 39% of sodium intake was consumed at dinner and 31% at lunch [43]. 

### 4.3. Contribution of Dietary Food Sources (Excluding Added Salt) to the ANIBES Population Daily Sodium Intakes: Groups and Subgroups by Age Group

We identified the five food groups that were the major sources of sodium consumed by the ANIBES study population, excluding table and cooking salt: meat and processed meat products (27%), cereals and grains (26%), milk and dairy products (14%), ready-to-eat meals (13%) and fish and shellfish (6%). The main subgroups from each of these food groups were sausages and other processed meat products, bread, cheeses and canned fish and shellfish.

Remarkably, for children and adolescents, meat and processed meat products as well as cereals and grains represented 27% of their total daily sodium intakes each, meaning that only those two food groups account for more than 50% of their sodium intakes. Conversely, the elder population had a higher contribution from the cereals and grains group (29%), and lower from meat and processed meat products. Milk and dairy products equally contributed to total sodium intakes amongst children and the elderly, while it was only slightly lower for adolescents and adults. Of concern, ready-to-eat meals were major sodium sources for adolescents and children, and much lower in adults and the elderly. Fish and shellfish products had a higher consumption amongst adults and specially the elderly. A recent study from Cuadrado-Soto et al. [45] in Spanish children aged 7–11 y also assessed the major sources of sodium from the diet by using a 3-day dietary record. Although the main aim of this research was identifying the degree of processing of these foods, their results may be comparable to our study. The average dietary sodium intake was 2026 mg/day (5.1 g salt/day) and the main sources were meat and meat products, cereals and grains, milk and dairy products, ready-to-eat and pre-cooked dishes—proportions which were similar to our findings—and sugars and sweets, which only accounted for 2% according to our data. However, results from the NHANES showed that for US children aged 6 to 18 y, the top ten food categories that contributed to almost half (48%) of their sodium intake included pizza, Mexican-mixed dishes, sandwiches, breads, cold meat cuts, soups, savoury snacks, cheese, plain milk, and poultry [43]. In the United Kingdom, Bruce et al. [46] assessed data from 21,108 British households between October 2008 and September 2009, using purchasing data from 44,372 food products (product description, product weight, annual purchases) and sodium values (mg/100 g) to obtain the major contributors to sodium intake. The largest contributors were table salt (23%), processed meats (18%), bread and bakery products (13%), dairy products (12%), and sauces and spreads (11%). They identified that more than one-third of sodium purchased (37%) was provided for by five food categories: bacon, bread, milk, cheese, and sauces. Although British dietary patterns greatly differ from Spanish, we acknowledge these findings are quite consistent with ours when identifying major sodium sources, except for the fact that sauces only represent 3% of the ANIBES study sodium intakes. The Austrian population [18], which showed the lowest sodium intakes across Europe according to published data, had similar food sources to our results. Data were collected from the Austrian Nutrition Survey 2014/2016, in 2018 adults between 18 and 64 y and assessed by use of 24-h recalls, on two non-consecutive days. Excluding the main contributors which were condiments and table salt, cereals and cereal products, meat and meat products and dairy products accounted for the greatest sodium intakes [18]. Therefore, it seems that globally high sodium intakes are mostly unrelated to the different dietary patterns (e.g., Mediterranean Diet vs. Western Diet).

Comparability of published studies is limited as different collection methods are used to determine sodium and salt intake: 24-h dietary recalls, dietary records or 24-h urine samples. In the previously mentioned study by the EC (2013), countries reporting the highest salt consumption used the 24-h urinary sodium excretion method, either alone or in combination with the 24-h dietary recall, whereas countries reporting the lowest salt intake only used dietary studies for the assessment [40]. The inclusion or exclusion of salt added during cooking or at the table is also a confounding factor. In addition, some studies do not record foods consumed outside the home (restaurants, fast-foods, etc.). It is also important to remark that frequency of consumption rather than sodium density of food products could play a key role in excess sodium intakes, therefore identification of major sources across the population could be more effective than only focusing on sodium-rich products. For instance, basic (staple) foods such as bread are amongst the main sodium contributors for most European populations [40,46,47], and in turn, processed meats which are denser in sodium than bread, contribute to the same proportion of sodium intakes amongst the ANIBES study population. The High Level Group on Nutrition and Physical Activity from the European Commission [40] identified four priority groups for action due to their salt contents but also to their frequency of consumption. These were bread, processed meats, cheese and ready-to-eat dishes. Initiatives for salt reduction introduced across the EU include agreements with food business operators or representative sectorial associations, awareness raising actions and monitoring approaches.

In Spain, bread is one of the major dietary contributors of sodium, not only due to its salt content but also and mainly, to its consumption frequency as it is a staple product [48], even though a sharp decline in its consumption has been observed in the last decades. For this reason, the AESAN established an agreement in 2004 with bread’s sectorial association (CEOPAN) for which bakers compromised to decrease sodium levels in flour by up to 20% in 4 years. In a cross-sectional study published in 2014, Perez-Farinós et al. assessed salt content in a representative sample of commercial and handmade breads (*n* = 1137) by analytical measures [49]. They found that the mean salt content was 2.08 g/100 g (SD: 0.32) with a minimum value of 0.30 g and a maximum of 3.33 g. In addition, they compared their results with data from 2008 (when voluntary bread reformulation practices started), to find salts levels remained stable. At present, there are other ongoing agreements such as the one between AESAN and the Spanish Confederation of Meat Retailers (CEDERCARNE) in which they committed to reduce 10% of the sodium in their products within 2 years [50]. Very recently (2019), an additional effort products has been launched through a voluntary agreement between the Spanish Health Administration and the food industry, in order to reformulate over 3000 commonly eaten products, and is planned to be achieved by the end of year 2020. Another important fact to emphasise is that the population is not always completely aware of the amounts of salt they consume [51]. It has been estimated that most dietary sodium comes from processed food and beverage products [52] where sodium is added by the food industry to enhance flavour, texture, etc. The role of food labelling is therefore fundamental to allow consumers to identify products which are reduced or high in sodium [53]. For this reason, the Regulation (EU) No. 1169/2011 on the provision of food information to consumers, adopted by the European Parliament and the Council in 2011, established that the term “salt” instead of “sodium” must be used on food labels to guarantee improved consumer understanding [54] as it is the major form in which sodium is added to foods and beverages.

### 4.4. Sodium Intakes According to Body Composition

According to our results regarding sodium intakes related to body composition of the ANIBES population, there were significant differences between sodium intakes of children who were overweight, as they showed higher intakes than those who were underweight. No differences were found for other age or BMI group. Results of the FANPE study in a representative sample of the adult Spanish population (*n* = 418, 18–60 y) [55] found that sodium intake adjusted by energy was related to weight gain promotion. They showed a positive association between BMI and urinary sodium concentration, as well as with waist circumference and waist/height ratio, which we did not observe amongst the ANIBES study individuals. Hasenegger et al. [18] also found a significant positive association between dietary salt intake and body weight (r = 0.186, *p* = 0.001), body height (r = 0.241, *p* = 0.001), body mass index (r = 0.082, *p* = 0.001) and waist circumference (r = 0.153, *p* = 0.001) in the Austrian population. Dietary salt intake was reported to be significantly lower (z = −3.640, *p* = 0.002) among normal weight (4.91 (IQ range: 2.89) g/day) compared to obese persons (5.80 (IQ range: 3.76) g/day). Authors allege the possibility that salt intake might promote the consumption of foods that lead to weight gain as salty foods are more palatable and encourage people to consume greater quantities of these foods, and likewise, that people with excess weight make worse food choices, especially those towards foods with high contents of sodium. It is indeed a complex association and there are scarce studies on the subject, but the latest research indicates that sodium might be an independent risk factor for obesity and that sodium might alter body fat metabolism, although mechanisms remain unknown [56,57]. 

### 4.5. Sodium Intake from Total Population Segmented by Spanish Nielsen Areas, Habitat Size and by Income Level and Age Groups from the ANIBES Study Population

We found no significant differences for sodium intakes between geographic zones in Spain, neither related to habitat size. Nevertheless, when assessing intakes in relation to income levels, adult and senior groups showed significantly lower sodium intakes at lower income levels (≤ 1000€) when compared to higher (≥ 2000€). The study conducted in the Austrian population did not show these differences or any other related to sodium intakes and affluence [18]. However, the study by Cappuccio et al. [58] that assessed sodium intake across the Italian population did find geographic and socioeconomic variations. The MINISAL-GIRCSI was a cross-sectional survey conducted in Italy and designed specifically to assess sodium and potassium intake in which 3857 participants, aged 39–79 years old, were randomly sampled in 20 regions. Authors described a significant north–south pattern of sodium excretion, as participants living in southern Italy (i.e., Calabria, Basilicata and Puglia) had a significantly higher sodium excretion than elsewhere. They also found a significantly higher salt intake in disadvantaged social groups (low-skill workers and lower education).

### 4.6. Strengths and Limitations

Several potential limitations exist in our analysis. First, dietary recall data may be subject to reporting error (where misreporting of intake may be particularly prevalent among children and adolescents). However, the methodology used to collect the presented data had the advantage of the use of mobile tablet technology to record food consumption, therefore overcoming limitations related to memory and bias. According to literature, underestimation of salt intakes is around 29% to 41% [23] due to underestimating and underreporting, difficulties in quantifying salt content of processed foods and discretionary salt use (in home, cooking or at the table) [37]. As already explained, the presented data do not include the discretionary salt added at the table or during cooking; therefore, the actual total salt intake is underestimated. Second, the results are limited by the cross-sectional design of the study, which does not allow judgements of causal relations, rather only associations. Third, dietary surveys are often considered unsuitable for estimating population sodium intake as they tend to underestimate intake due to the variability of the salt content in recipes for both processed and home-cooked foods and the difficulty to quantify discretionary salt use. Indeed, the gold standard for assessing total sodium intake is 24-h urine sodium excretion.

Food composition tables and databases also hold important limitations, as there is a lack of updated and comprehensive data on processed foods and ready-to-eat foods as this requires considerable effort [9]. In addition, there are many ways of aggregating individual foods into food groups and subgroups; in the present study, classification was made according to previous ANIBES studies [33] that recently updated the food groups and subgroups of the Spanish market, a key strength in the present study.

## 5. Conclusions

Reducing the dietary sodium intake is a challenging and relevant public health goal as intakes are above recommendations for all age groups, mainly for the youngest, and for both genders. Sodium reduction is a relevant, cost-effective strategy to reduce high blood pressure and help prevent cardiovascular disease, the leading cause of death in Spain and throughout the world. The major sodium food sources within the Spanish diet are meat and meat products, cereals and grains, milk and dairy products and ready-to-eat meals. Therefore, improving the quality of nutritional composition of foods as well as moderating certain groups consumption are key goals for public health authorities and for achieving a balanced diet in the Spanish population. Consequently, the data presented may shape and inform public health policies aimed at reducing the sodium content of the diet.

## Figures and Tables

**Table 1:** Sodium intake from foods consumed by the ANIBES study population (excluding added table and cooking salt).

			*N*	Total Sodium Intake (mg/day)
**Total Population (9–75 y)**		Total	2009	2025 ± 805
		Men	1013	2218 ± 868 *
		Women	996	1828 ± 682
**Age**	9–12 y	Total	213	2247 ± 735 ^a^
		Men	126	2350 ± 798 *
		Women	87	2098 ± 608
	13–17 y	Total	211	2351 ± 842 ^a^
		Men	137	2517 ± 868 *
		Women	74	2043 ± 698
	18–64 y	Total	1655	2026 ± 805 ^b^
		Men	798	2219 ± 876 *
		Women	857	1846 ± 686
	65–75 y	Total	206	1693 ± 640 ^c^
		Men	99	1841 ± 668 *
		Women	107	1556 ± 583

Data reported as means ± standard deviation (SD) per group. Different superscript lowercase letters indicate statistically significant difference between ages (all differences are *p* < 0.001; Kruskal–Wallis test) and * indicate statistically significant difference between gender, (all differences are *p* ≤ 0.05; Mann–Whitney U test). ANIBES, “anthropometric data, macronutrients and micronutrients intake, practice of physical activity, socioeconomic data and lifestyles in Spain”.

**Table 2:** Sodium intakes from foods per 1000 kcal consumed by the ANIBES study population (excluding added table and cooking salt).

			*N*	Sodium (mg/day) Per 1000 kcal
**Total Population (9–75 y)**		Total	2009	1118 ± 315
		Men	1013	1130 ± 307
		Women	996	1106 ± 322
**Age**	9–12 y	Total	213	1146 ± 265 ^a^
		Men	126	1169 ± 266
		Women	87	1113 ± 263
	13–17 y	Total	211	1166 ± 303 ^a^
		Men	137	1190 ± 304
		Women	74	1122 ± 299
	18–64 y	Total	1655	1116 ± 317 ^a,b^
		Men	798	1125 ± 306
		Women	857	1109 ± 327
	65–75 y	Total	206	1052 ± 299 ^b^
		Men	99	1048 ± 307
		Women	107	1056 ± 294

Data reported as means ± standard deviation (SD) per group. Values that do not share superscript are significantly different between ages, *p* ≤ 0.05. (Kruskal–Wallis test).

**Table 3:** Sodium intakes from foods consumed at different eating occasions by age groups from the ANIBES study population (excluding added table and cooking salt).

Sodium Intakes by Eating Occasion/Period (mg/day).	Children	Adolescents	Adults	Elderly
	9–12 Years Old	13–17 Years Old	18–64 Years Old	65–75 Years Old
**n**	213	211	1655	206
**Breakfast**	376.4 ± 188.6	363.9 ± 229.9	309.6 ± 246.0	305.9 ± 203.6
**Mid-morning**	246.8 ± 266.6	278.6 ± 335.6	111.6 ± 225.8	61.8 ± 156.6
**Lunch**	569.7 ± 278.7	623.1 ± 382.2	692.6 ± 406.3	635.2 ± 341.5
**Afternoon**	309.6 ± 260.1	202.3 ± 244.8	101.1 ± 186.3	75.9 ± 142.2
**Dinner**	695.6 ± 373.7	824.9 ± 487.9	756.6 ± 453.1	599 ± 360.3
**Other occasions**	48.9 ± 112.2	58 ± 184.8	54.1 ± 141.7	15.1 ± 48.1
**Working days**	2348.8 ± 883.7	2382.5 ± 998.8	2014.5 ± 907.3	1712.9 ± 729.3
**Non-working days**	2043.4 ± 1021.1	2287.1 ± 1092.2	2047.6 ± 1129.6	1653 ± 880.7

Data reported as means ± standard deviation (SD) per group.

**Table 4:** Dietary sources of sodium from food groups and subgroups consumed by the ANIBES study population (9–75 y).

Food Groups	Food Subgroups	Sodium Contribution (mg/day)	Sodium Contribution (%)
**Meat and meat products**		530.3 ± 410.0	27
	Sausages and other processed meats products	403.8 ± 371.9	
	Fresh meat	125.0 ± 157.5	
	Viscera and offal	1.5 ± 9.2	
**Cereals and Grains**		522.0 ± 271.1	26
	Bread	400.4 ± 244.4	
	Bakery and pastry	84.3 ± 108.2	
	Breakfast cereals and cereal bars	24.7 ± 71.2	
	Grains and flours	4.7 ± 13.7	
	Tubers	4.4 ± 10.4	
	Pasta	3.6 ± 24.0	
**Milk and dairy products**		280.5 ± 232.4	14
	Cheese	150.0 ± 209.2	
	Milk	88.9 ± 70.2	
	Yogurt and fermented milk	29.3 ± 40.2	
	Other dairy products	12.3 ± 29.4	
**Ready-to-eat meals**		261.9 ± 330.2	13
**Fish and shellfish**		120.1 ± 198.4	6
	Canned fish and shellfish	74.1 ± 182.3	
	Shellfish	23.4 ± 56.3	
	Fish	23.4 ± 33.9	
**Appetisers**		77.1 ± 168.0	4
**Sauces and condiments**		59.5 ± 125.1	3
**Vegetables**		49.1 ± 49.7	2
**Sugars and sweets**		37.9 ± 70.9	2
	Chocolates	37.3 ± 70.9	
	Jams and similar	0.4 ± 1.3	
	Sugars	0.2 ± 0.6	
**Eggs**		36.4 ± 39.1	2

Data reported as means ± standard deviation (SD), per group.

**Table 5:** Sodium intakes according to body composition and physical activity amongst the ANIBES study population.

		Sodium Intakes (mg/d)				
		Total Population	Children 9–12 y	Adolescents 13–17 y	Adults 18–64 y	Elderly 65–75 y
**BMI**	**Underweight**	2245.7± 771.5	2252.3 ± 681.2	2327.1 ± 793.8	2151.1 ± 983.8	-
		*n* = 143	*n* = 85	*n* = 28	*n* = 30	
	**Normal**	2129.3 ± 840.5	2205.1 ± 779.0	2392.9 ± 905.9	2084.4 ± 827.2	1725.6 ±733.1
		*n* = 1000	*n* = 112	*n* = 146	*n* = 704	*n* = 38
	**Overweight**	1953.8 ± 745.9	2543.0 ± 694.4 *	2177.4 ± 561.7	1970.2 ± 761.4	1694.9 ± 619.5
		*n* = 740	*n* = 15	*n* = 33	*n* = 592	*n* = 100
	**Obesity**	1938.6 ± 788.7	-	2404.0 ± 688.6	1987.8 ± 810.6	1671.7 ± 622.7
		*n* = 402		*n* = 4	*n* = 329	*n* = 68
**Waist/Height**	**No risk**	2174.8 ± 845.2	2247.9 ± 761	2387.1 ± 876.2	2107.1 ± 846.2	1842.5 ± 831.7
		*n* = 1043	*n* = 162	*n* = 181	*n* = 689	*n* = 11
	**Risk**	1938.3 ± 750.1	2243.9 ± 654.3	2131.3 ± 554.9	1967.4 ± 769.9	1684.5 ± 629.0
		*n* = 1242	*n* = 51	*n* = 30	*n* = 966	*n* = 195

(-): not determined. Data reported as means ± standard deviation (SD) per group. * *p* < 0.05 compared to underweight (Kruskal–Wallis test).
